# Alkaline Phosphatase Treatment of Acute Kidney Injury in an Infant Piglet Model of Cardiopulmonary Bypass with Deep Hypothermic Circulatory Arrest

**DOI:** 10.1038/s41598-019-50481-w

**Published:** 2019-10-02

**Authors:** Jesse A. Davidson, Ludmila Khailova, Amy Treece, Justin Robison, Danielle E. Soranno, James Jaggers, Richard J. Ing, Scott Lawson, Suzanne Osorio Lujan

**Affiliations:** 1 University of Colorado Denver, Department of Pediatrics, Auroa, CO 80045 USA; 2 University of Colorado Denver, Department of Pathology, Auroa, CO 80045 USA; 3 University of Colorado Anschutz Medical Campus, Department of Surgery, Auroa, CO 80045 USA; 4 University of Colorado Anschutz Medical Campus, Department of Anesthesiology, Auroa, CO 80045 USA; 5 Children’s Hospital Colorado, Heart Institute, Aurora, CO 80045 USA

**Keywords:** Diagnostic markers, Cardiology, Paediatric research, Preclinical research, Translational research

## Abstract

Acute kidney injury (AKI) is associated with prolonged hospitalization and mortality following infant cardiac surgery, but therapeutic options are limited. Alkaline phosphatase (AP) infusion reduced AKI in phase 2 sepsis trials but has not been evaluated for cardiac surgery-induced AKI. We developed a porcine model of infant cardiopulmonary bypass (CPB) with deep hypothermic circulatory arrest (DHCA) to investigate post-CPB/DHCA AKI, measure serum/renal tissue AP activity with escalating doses of AP infusion, and provide preliminary assessment of AP infusion for prevention of AKI. Infant pigs underwent CPB with DHCA followed by survival for 4 h. Groups were treated with escalating doses of bovine intestinal AP (1, 5, or 25U/kg/hr). Anesthesia controls were mechanically ventilated for 7 h without CPB. CPB/DHCA animals demonstrated histologic and biomarker evidence of AKI as well as decreased serum and renal tissue AP compared to anesthesia controls. Only high dose AP infusion significantly increased serum or renal tissue AP activity. Preliminary efficacy evaluation demonstrated a trend towards decreased AKI in the high dose AP group. The results of this dose-finding study indicate that AP infusion at the dose of 25U/kg/hr corrects serum and tissue AP deficiency and may prevent AKI in this piglet model of infant CPB/DHCA.

## Introduction

Approximately 25% of infants with congenital heart disease (CHD) require invasive treatment in the first year of life. Cardiopulmonary bypass (CPB) is utilized for most neonatal and infant cardiac surgeries to maintain non-thoracic organ perfusion during the repair. Additional techniques such as deep hypothermic circulatory arrest (DHCA) and selective cerebral perfusion (SCP), which result in cessation of all blood flow to the thorax and abdomen, are often required for complex repairs. While use of these techniques is necessary for successful repair, these strategies also contribute directly to systemic derangements in the perioperative period, including splanchnic hypoperfusion, ischemia-reperfusion injury, the systemic inflammatory response syndrome, and post-operative organ dysfunction^1–5^. These physiologic derangements complicate recovery and lead to increased post-operative morbidity and mortality^6,7^.

Acute kidney injury (AKI) is an increasingly recognized and important complication of pediatric cardiac surgery^8^. The etiology of AKI in this population is complex, but both direct and indirect physiologic effects of cardiac surgery, CPB, and DHCA/SCP are known contributors^9,10^. Evidence of proximal tubular injury appears within 2 hours of cardiac surgery in both animal models and human biomarker studies^11–14^, followed by evolution to clinical acute kidney dysfunction over a period of hours to days^15^. Incidence of AKI following pediatric cardiac surgery ranges from 25–64% depending on the specific population studied and definition used^11,16–18^, with an associated mortality between 5–12%^19,20^. Development of AKI is also associated with increased duration of mechanical ventilation and length of hospital stay and may increase risk for development of chronic kidney disease^6,21,22^. While substantial progress has been made in identifying early AKI using novel kidney injury biomarkers, options to prevent or treat AKI remain largely limited to supportive care and avoidance of nephrotoxic medications. Novel therapies to decrease the incidence and severity of AKI following cardiac surgery are greatly needed.

Alkaline phosphatases (AP) are endogenous metalloenzymes found in serum and in multiple organs throughout the body including bone, liver, intestine, and kidney^23^. These enzymes are well established as biomarkers of liver and bone disease, but their physiologic roles remain incompletely understood. Recent evidence points towards a potential protective effect of AP in the mitigation of AKI through dephosphorylation of nephrotoxic molecules including extracellular adenine nucleotides^24–26^ and endotoxin^27–29^. Phase 2 human studies have demonstrated efficacy of AP in the treatment of sepsis-induced AKI^30–32^. Less is known, though, about the role of AP as a potential therapeutic agent for cardiac surgery-induced AKI. Serum AP activity decreases in adult and pediatric patients during cardiac surgery^33–36^ and lower post-operative serum AP activity is independently associated with higher peak post-operative creatinine^37^. In a phase 2 study of adults undergoing coronary artery bypass grafting, perioperative infusion of bovine AP was well tolerated and decreased post-operative cytokine expression^38^. To our knowledge, however, there are no preclinical or human studies evaluating dosing or preliminary efficacy of systemic AP infusion for prevention of CPB/DHCA-induced AKI.

In this study we sought to establish a porcine model of early infant CPB/DHCA-induced AKI and determine the serum and renal tissue AP activity achieved with escalating doses of bovine intestinal AP (BiAP) infusion in this model. As a secondary aim, we sought to determine optimal dosing and preliminary efficacy data for BiAP infusion to reduce early AKI in this model. We hypothesized that our model would result in AKI as determined by increased histologic and biomarker evidence of proximal tubular damage. We further hypothesized that continuous infusion of BiAP would raise serum and renal tissue AP activity *in vivo* in a dose-dependent manner and would not significantly alter cardiovascular physiology. Finally, we hypothesized that higher doses of BiAP would result in decreased histologic and biomarker evidence of AKI in this model.

## Results

### Evidence of shock and AKI in the CPB/DHCA piglet model

#### Clinical Physiology

As an initial analysis, we combined data from all animals undergoing CPB/DHCA regardless of treatment group and compared them to anesthesia-only controls in order to determine the cardiovascular effects of the model as well as the level of AKI produced by the model. At baseline (following induction of anesthesia), there was no difference in heart rate, systolic or mean arterial blood pressure, or base deficit between CPB animals and anesthesia controls (Supplemental Figure S1). As expected, CPB with DHCA consistently produced cardiovascular shock in our animals similar to that seen in infants undergoing cardiac surgery. CPB/DHCA animals demonstrated a significantly higher heart rate distribution and lower base deficit distribution compared to anesthesia controls (Supplemental Figure S1) from rewarming through euthanasia. No difference was found in systolic or mean arterial blood pressure between CPB animals and controls due to the titration of inotropic support to target similar blood pressures (Supplemental Figure S1). CPB/DHCA animals also showed higher lactate distribution (median 3.42 mmol/L (range 0.38–7.94) vs 0.65 mmol/L (range 0.37–0.66); p = 0.0006) (Supplemental Figure S2) and vasoactive inotropic score (VIS)^39,40^ distribution (median 8.85 (range 7–26) vs 0 (range 0–0); p < 0.0001) (Supplemental Figure S3) prior to euthanasia compared to anesthesia controls.

#### Kidney histology score

**Figure 1:**
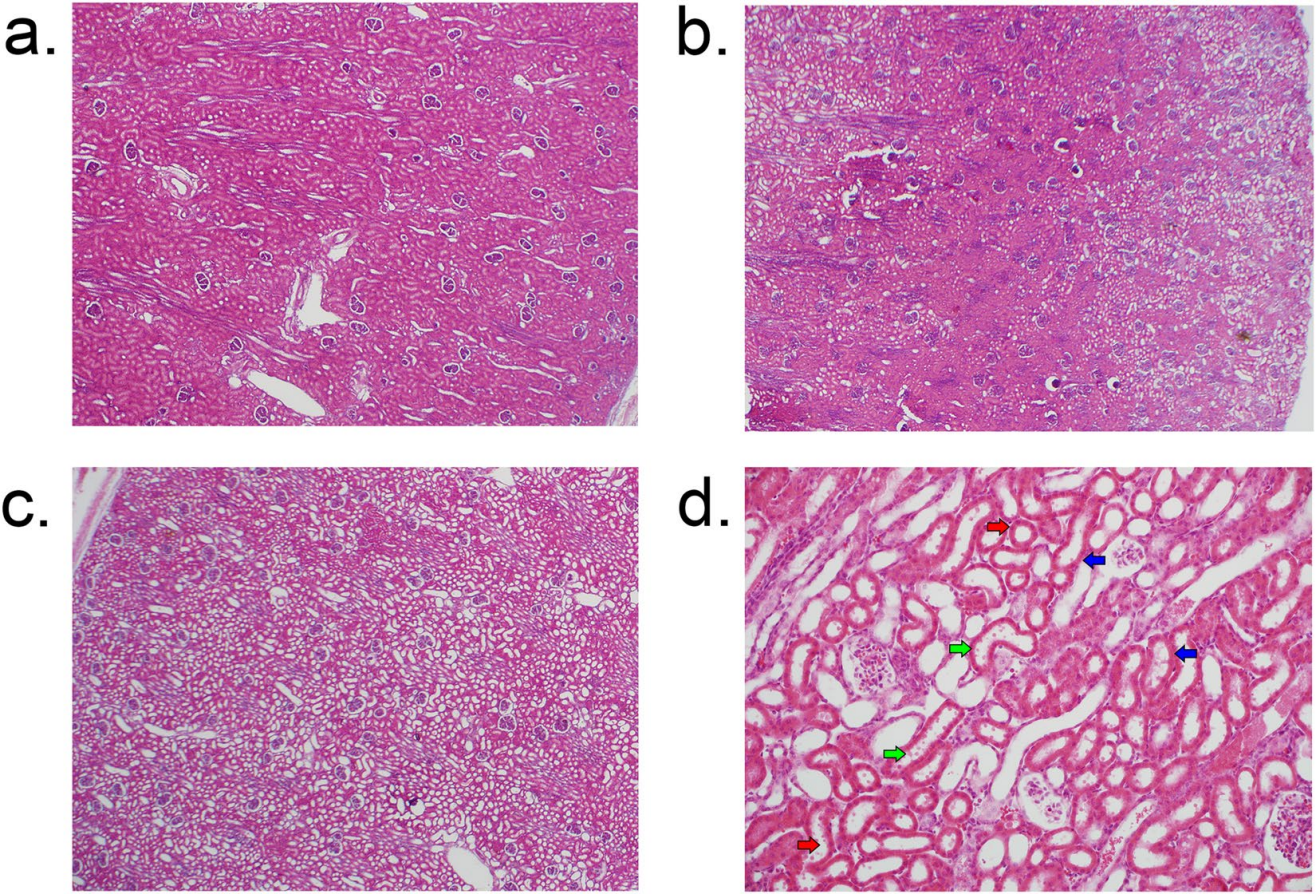
Hematoxylin-eosin staining of renal cortex for acute kidney injury histology scoring. (**a**) Representative section from an anesthesia control animal demonstrating normal histology without evidence of AKI on low magnification (4x); (**b**) Section from an animal 4 hours after CPB/DHCA demonstrating moderate AKI with 25–50% of cortical area affected; (**c**) Section from an animal four hours after CPB/DHCA demonstrating severe AKI with 75–100% of cortical area affected; (**d**) high-magnification (20x) view of damaged proximal tubules demonstrating proximal tubular dilation (red arrows), epithelial thinning (blue arrows), and epithelial blebs (green arrows). CPB/DHCA = cardiopulmonary bypass with deep hypothermic circulatory arrest; AKI = acute kidney injury.

**Figure 2:**
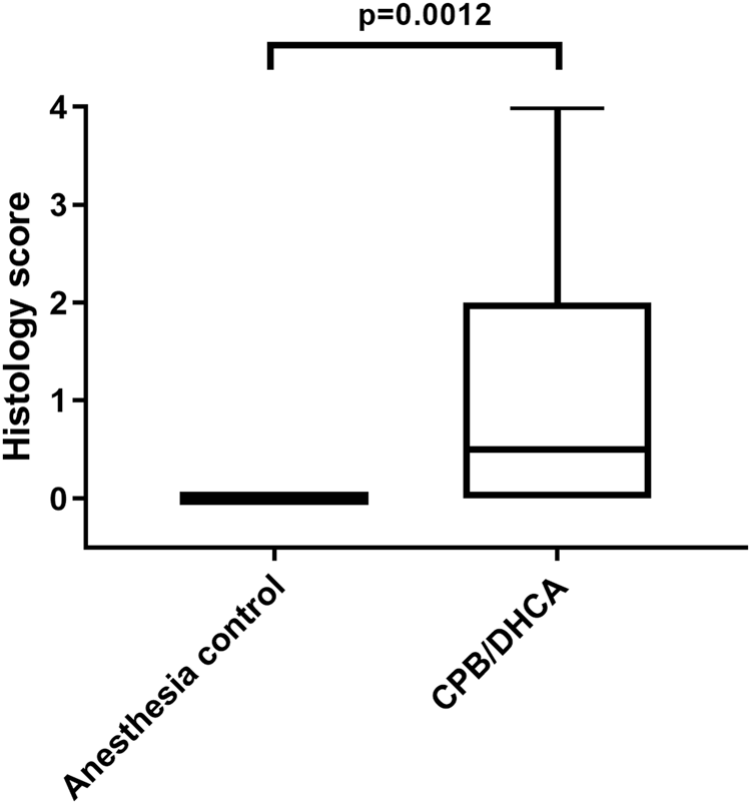
Distribution of AKI histology scoring in animals undergoing CPB/DHCA versus anesthesia controls. AKI scoring based on percent affected cortical area on low magnification examination: 0 = none to less than 5%, 1 = 5–25%, 2 = 26–50%, 3 = 51–75%, 4 = greater than 75%. CPB/DHCA = cardiopulmonary bypass with deep hypothermic circulatory arrest; AKI = acute kidney injury.

On low magnification examination, kidneys from anesthesia controls were consistently normal (Fig. 1A**)**. Conversely, kidneys of CPB/DHCA animals demonstrated a range of AKI findings from minimal injury to severe diffuse AKI with proximal tubular dilatation and epithelial flattening (Fig. 1B,C). High magnification views of kidneys with AKI demonstrated damaged proximal tubules with tubular dilation, epithelial flattening and epithelial blebs (Fig. 1D). The histologic kidney injury score distribution of CPB/DHCA animals was significantly higher than anesthesia controls (Fig. 2).

#### Hemodynamics and AKI

There were no significant differences in the distribution of hemodynamic variables between animals with and without AKI (VIS: p = 0.31; heart rate: p = 0.74; systolic blood pressure: p = 0.59; mean arterial blood pressure: p = 0.46; lactate: p = 0.73). Similarly, we found no significant differences in the distribution of speed of cooling (p = 0.60) or rewarming (p = 0.83) by Mann-Whitney U test in animals with and without AKI.

#### Biomarkers of AKI

**Figure 3:**
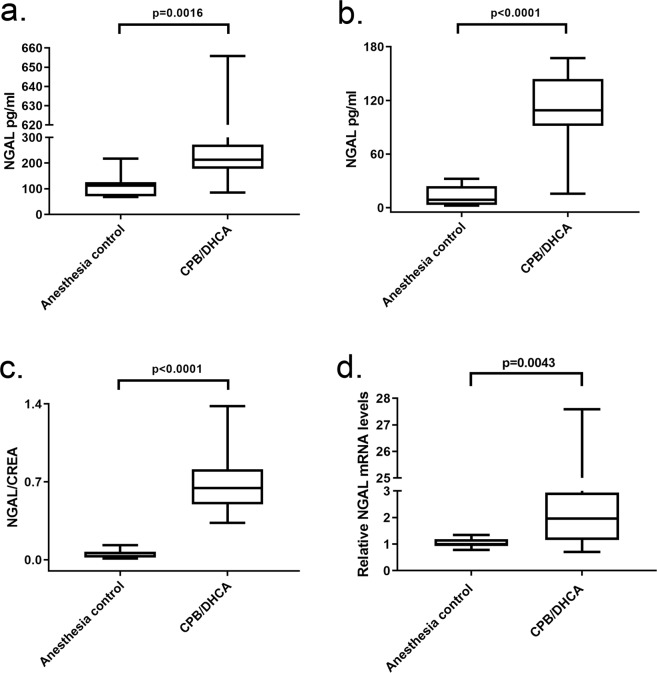
Comparison of distribution of AKI biomarkers between animals undergoing CPB/DHCA vs anesthesia controls. Serum and urine samples obtained immediately prior to euthanasia. (**a**) serum NGAL; (**b**) urine NGAL; (**c**) urine NGAL/creatinine ratio; (**d**) tissue NGAL mRNA expression. AKI = acute kidney injury; CPB/DHCA = cardiopulmonary bypass with deep hypothermic circulatory arrest; NGAL = neutrophil gelatinase-associated lipocalin.

The distribution of serum creatinine at euthanasia was not different between the CPB/DHCA group (median 1.1 mg/dl (range 0.7–2.4)) and anesthesia controls (median 1.1 mg/dl (range 0.7–1.9)) (p = 0.84). CPB/DHCA animals did demonstrate significantly increased serum neutrophil gelatinase-associated lipocalin (NGAL) levels (Fig. 3A) as well as urine NGAL and NGAL/creatinine ratios compared to anesthesia controls (Fig. 3B,C). Consistent with serum and urine analysis, the distribution of kidney tissue NGAL messenger RNA (mRNA) levels was significantly higher in the CPB/DHCA group compared to anesthesia controls (Fig. 3D). Interestingly, the distribution of kidney injury molecule-1 (KIM-1) mRNA levels was significantly lower in animals undergoing CPB/DHCA than in animals undergoing anesthesia only (0.4 (range 0.09–1.42) vs 1.0 (range 0.045–2.02); p = 0.006) (Supplemental Figure S4). The distribution of interleukin-6 (IL-6) mRNA levels trended higher in CPB/DHCA animals but this difference did not reach statistical significance (4.7 (range 0.27–15.44 vs 1 (range 0.15–9.65); p = 0.16) (Supplemental Figure S5).

Biomarkers of early AKI showed mixed associations with kidney injury as assessed by histology scoring. The distribution of serum creatinine at euthanasia (4 hours post-CPB) was not different in animals with and without histologic evidence of AKI (median 1.1 mg/dl (range 0.7–2.4) vs 1.1 mg/dl (range 0.7–1.9); p = 0.37) (Supplemental Figure S6), an unsurprising finding given the early stage of ischemia-reperfusion injury in this study. Serum NGAL trended higher in animals with AKI but this finding did not reach statistical significance (median 204 ng/ml (range 68–656) vs 167 ng/ml (range 164–443); p = 0.11) (Supplemental Figure S7). There was a nonsignificant trend towards differential distribution of NGAL mRNA by severity of AKI (mild-to-moderate injury: median 2.1-fold increase (range 1–27.6); severe injury 1.4-fold increase (range 0.7–6.2); no injury 1.4-fold increase (range 0.8–8.2); p = 0.13 for a difference among groups) (Supplemental Figure S8). As expected, urine NGAL/creatinine ratios were significantly higher in injured vs non-injured kidneys (0.70 (range 0.45–1.36) vs 0.37 (range 0.01–1.38); p = 0.02) (Supplemental Figure S9). Surprisingly, KIM-1 mRNA was markedly lower in injured kidney tissue compared to non-injured tissue (0.26 (range 0.09–0.79) vs. 0.64 (range 0.21–2.02); p = 0.0006) (Supplemental Figure S10).

#### Tissue AP activity and kidney histology score

**Figure 4:**
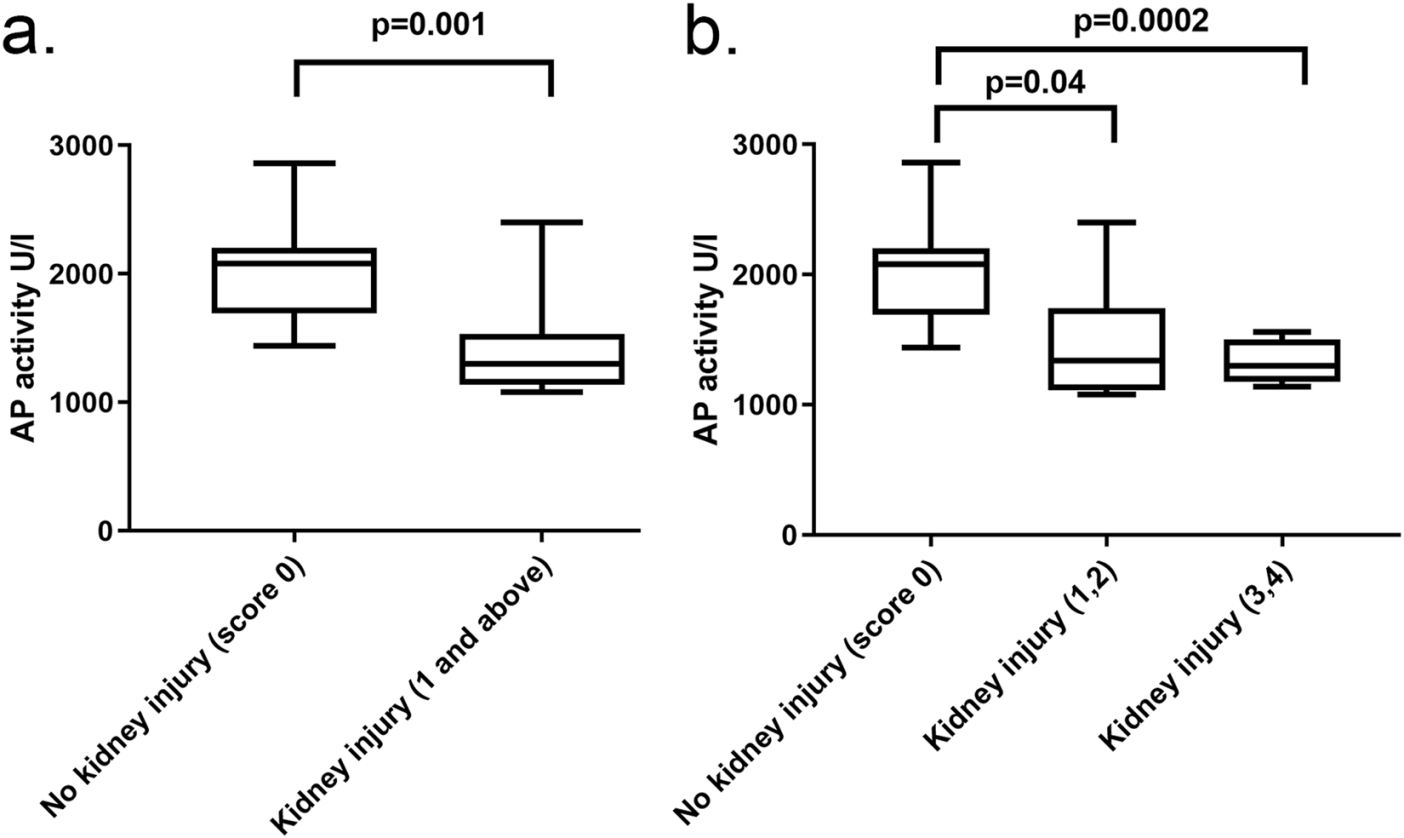
Comparison of the distribution of tissue AP activity by kidney injury histology score. AKI scoring based on percent affected cortical area on low magnification examination: 0 = none to less than 5%, 1 = 5–25%, 2 = 26–50%, 3 = 51–75%, 4 = greater than 75%. (**a**) AKI score 0 versus AKI score 1–4; (**b**) AKI score 0 versus 1–2 versus 3–4. AP = alkaline phosphatase; AKI = acute kidney injury.

We next evaluated tissue AP activity compared to kidney injury histologic scoring. Animals with at least mild AKI (score 1–4) demonstrated significantly lower tissue AP activity compared to animals with no AKI (Fig. 4A). Animals with severe AKI (score 3–4) showed a more consistent decrease in tissue AP activity with a trend towards a dose-response between tissue AP activity and severity of histologic AKI (Fig. 4B).

### Serum AP kinetics and tissue AP activity

**Figure 5:**
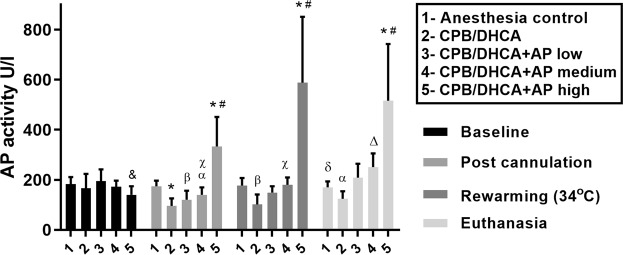
Serum AP activity at baseline, following cannulation to cardiopulmonary bypass, at rewarming, and at euthanasia. Comparisons for differences in mean serum AP activity between interventional groups at each time point are presented. CPB/DHCA = cardiopulmonary bypass with deep hypothermic circulatory arrest; AP = alkaline phosphatase; * p < 0.005 versus anesthesia controls; # p < 0.01 versus CPB/DHCA and CPB/DHCA + AP low; & p < 0.05 versus anesthesia controls; α p < 0.05 vs CPB/DHCA + AP low; β p < 0.01 versus anesthesia controls; χ p < 0.05 versus CPB/DHCA; δ p < 0.05 versus CPB/DHCA; Δ p < 0.05 versus CPB/DHCA + AP high.

Serum AP activity was measured at induction, cannulation, rewarming and euthanasia (4 hours post-bypass). There were no significant differences in AP activity between intervention groups at baseline (induction of anesthesia) (Fig. 5). Groups were significantly different by one-way ANOVA at all remaining time points. Group differences were driven by a significantly lower mean serum AP activity in the CPB/DHCA without AP group compared to anesthesia controls (post cannulation (95 ± 30 vs 175 ± 22U/l; p = 0.002), rewarming (102 ± 39 vs 177 ± 30U/l; p = 0.008), and euthanasia (124 ± 30 vs 170 ± 24U/l; p = 0.02)) as well as higher AP activity in the CPB/DHCA with high dose AP group (25U/kg/h) compared to either anesthesia controls (post cannulation (334 ± 118 vs 175 ± 22 U/L; p = 0.003), rewarming (588 ± 263 vs 177 ± 30 U/L; p = 0.002) and euthanasia (516 ± 227 vs 170 ± 24 U/L; p = 0.003)) or animals undergoing CPB/DHCA without AP (post cannulation (334 ± 118 vs 95 ± 30 U/L; p = 0.008), rewarming (588 ± 263 vs 102 ± 39 U/L; p = 0.009) and euthanasia (516 ± 227 vs 124 ± 30 U/L; p = 0.007)). The initial decrease in AP in animals undergoing CPB/DHCA without AP infusion began immediately after initiation of CPB due to poor-AP activity in the adult porcine whole blood/crystalloid CPB prime (mean 35U/l (SD 25)) compared to pre-CPB serum from the piglets (167 U/L (SD 45)) (p < 0.0001). Low dose (1U/kg/h) as well as medium dose (5U/kg/h) BiAP infusion did not significantly increase serum AP activity at any time point when compared to anesthesia controls or CPB/DHCA animals without BiAP infusion.

**Figure 6:**
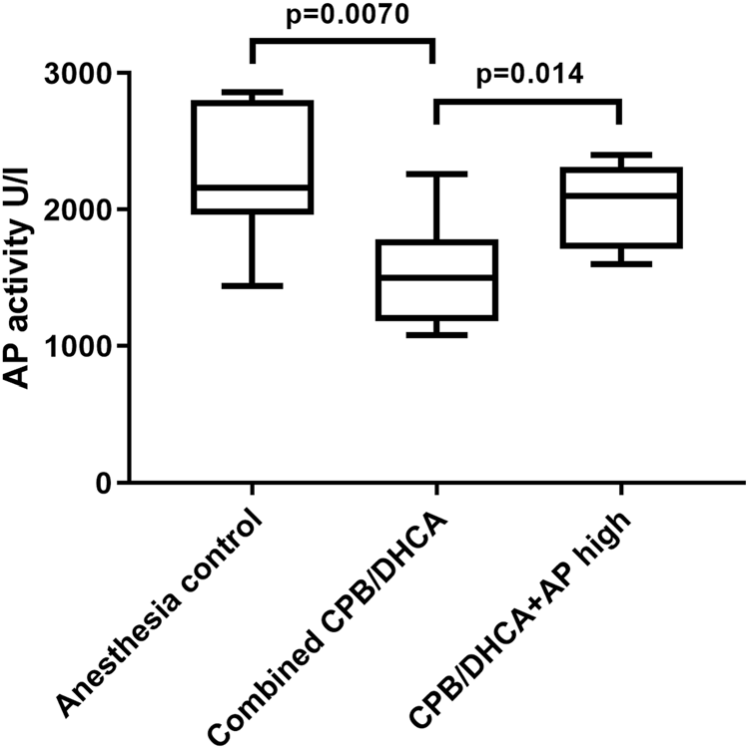
Comparison of the distribution of tissue AP activity by intervention group (anesthesia controls vs combined no AP infusion/low dose AP infusion/medium dose AP infusion vs high dose AP infusion). CPB/DHCA = cardiopulmonary bypass with deep hypothermic circulatory arrest; AP = alkaline phosphatase.

Tissue AP activity also did not increase with either the low dose (median tissue AP activity 1500 U/L, range 1080–2260) or medium dose (median tissue AP activity 1480 U/L, range 1180–1560) AP infusions compared to CPB/DHCA animals with no AP infusion (median 1780U/L, range 1120–2140) (p = 0.88). Kidney tissue AP activity was significantly lower in the combined group of CPB/DHCA animals with no AP, low dose AP, or medium dose AP infusions compared to anesthesia controls (Fig. 6). High dose BiAP infusion led to a significant increase in tissue AP activity compared to the remaining combined CPB/DHCA animals. Tissue AP activity in the high dose BiAP group was comparable to anesthesia controls (2100 U/L (range 1600–2400) vs 2160 U/L (range 1440–2860); p = 0.76).

Overall, low and medium dose BiAP infusions failed to significantly change serum or tissue AP activity, while both serum and tissue AP activity were substantially increased with high dose BiAP infusion. Therefore, subsequent analyses were performed combining the no BiAP infusion, low dose BiAP infusion, and medium dose BiAP infusion groups (“combined CPB/DHCA”; n = 15) for comparison versus the high dose BiAP group (n = 5) and anesthesia only control group (n = 7) in order to evaluate the effect of high dose BiAP on physiology and AKI injury in our model.

### High dose AP and cardiovascular physiology

At 4 hours post bypass, high dose BiAP animals demonstrated higher lactate, VIS, and HR than anesthesia controls (Supplemental Figure S11). Lactate, VIS, and HR in the high dose BiAP group were not significantly different from the remaining combined CPB/DHCA group, providing preliminary evidence that high dose BiAP infusion does not markedly change cardiovascular physiology after CPB/DHCA.

### Effect of high dose AP on AKI

#### Kidney histology score by treatment group

**Figure 7:**
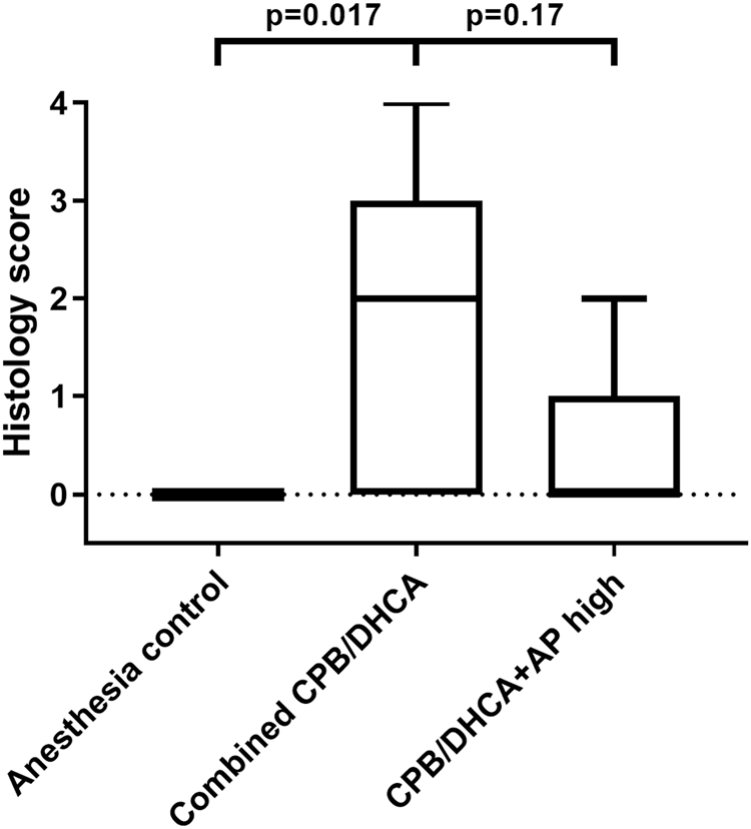
AKI histology score by intervention group (anesthesia controls vs combined no AP infusion/low dose AP infusion/medium dose AP infusion vs high dose AP infusion). AKI scoring based on percent affected cortical area on low magnification examination: 0 = none to less than 5%, 1 = 5–25%, 2 = 26–50%, 3 = 51–75%, 4 = greater than 75%. CPB/DHCA = cardiopulmonary bypass with deep hypothermic circulatory arrest; AP = alkaline phosphatase.

We first evaluated our primary outcome (histologic kidney injury score) among animals receiving high dose BiAP infusion, the remaining combined CPB/DHCA groups, and the anesthesia controls. Incidence of any histologic AKI (AKI score 1–4) was 20% (1/5) in the high dose BiAP group compared to 60% (9/15) in the remaining CPB/DHCA groups and 0% (0/7) in the anesthesia control group (p = 0.019 for a difference among groups). Incidence of severe AKI (AKI score 3–4) was 0% (0/5) in the high dose group, 27% (4/15) in the remaining CPB/DHCA groups, and 0% (0/7) in the anesthesia control group (p = 0.26 for a difference among groups). Histology scores differed significantly among the high dose AP group, remaining combined CPB/DHCA groups, and anesthesia controls, driven by the higher AKI score in the combined CPB/DHCA group (Fig. 7). There was no significant difference between the histology scores of the high dose group and anesthesia controls (p = 0.42).

#### Biomarkers of AKI at 4 hours post CPB/DHCA

**Figure 8:**
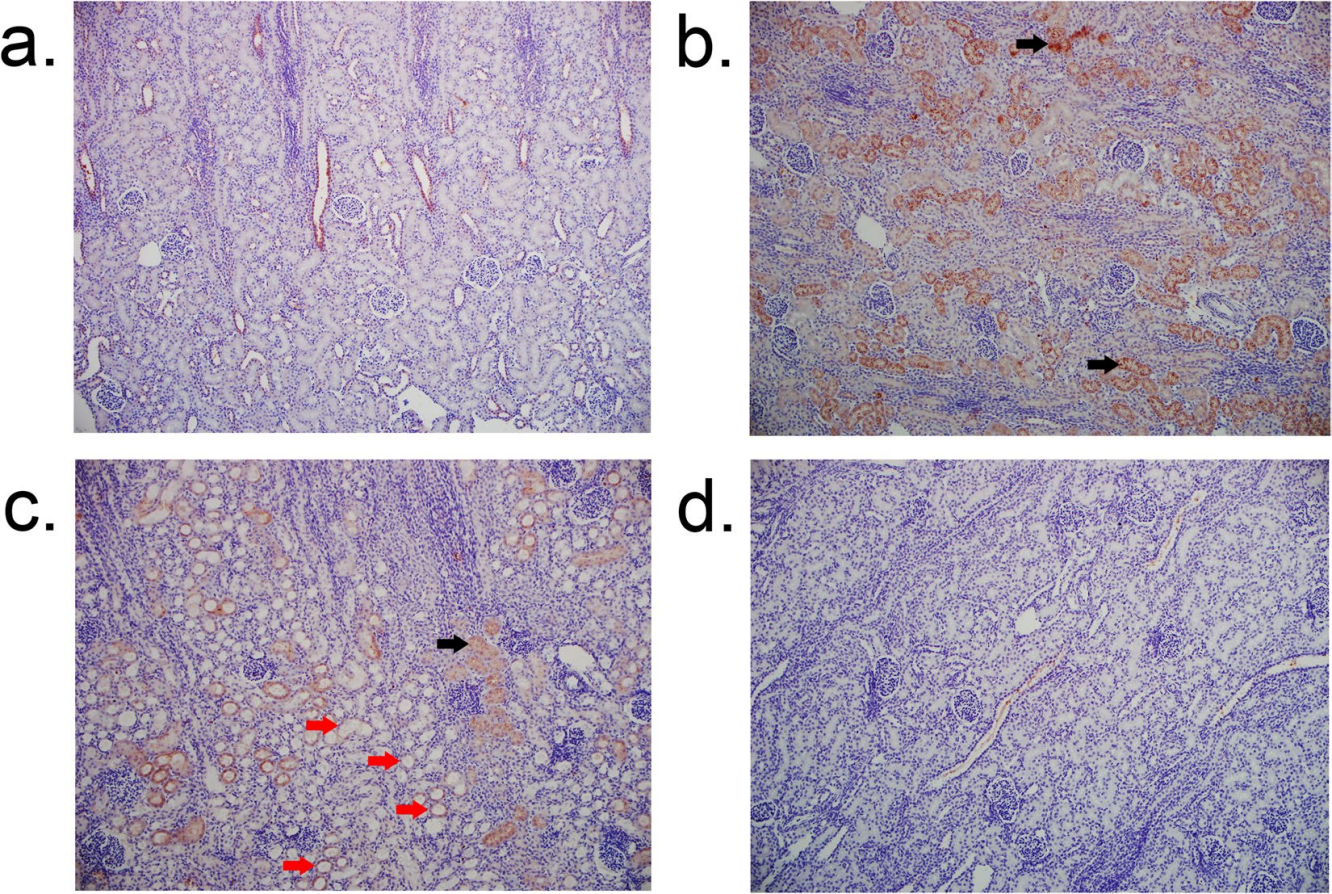
NGAL immunohistochemistry. (**a**) Anesthesia control demonstrating no significant NGAL staining; (**b**) and (**c**) CPB/DHCA demonstrating diffuse staining of histologically normal appearing proximal tubules and decreased staining in injured proximal tubules; (**d**) CPB/DHCA with high dose AP demonstrating minimal NGAL staining similar to anesthesia controls. Black arrows = prominent NGAL staining of histologically normal proximal tubules; red arrows = decreased NGAL staining in injured proximal tubules; CPB/DHCA = cardiopulmonary bypass with deep hypothermic circulatory arrest; NGAL = neutrophil gelatinase-associated lipocalin.

Serum NGAL, urine NGAL, and urine NGAL/creatinine ratios were elevated in animals undergoing CPB/DHCA compared to anesthesia controls, and we found no significant difference in serum NGAL (216 ng/ml (range 164–278) vs 196 ng/ml (range 86–656); p = 0.87) (Supplemental Figure S12), urine NGAL (109 ng/ml (range 16–131) vs 109 ng/ml (range 67–167); p = 0.45) (Supplemental Figure S13), or urine NGAL/Creatinine ratio (0.61 (range 0.33–1.38) vs 0.65 (range 0.37–1.36); p = 0.60) (Supplemental Figure S14) between the high dose AP group and the remaining CPB/DHCA groups. Similarly, no statistical differences were found in NGAL (2.7 (range 1.8–27.6) vs 1.8 (range 0.7–8.2); p = 0.10) (Supplemental Figure S15), KIM-1 (0.49 (range 0.24–1.42) vs 0.37 (range 0.1–1.3); p = 0.34) (Supplemental Figure S16), or IL-6 (6.8 (range 0.38–15.4) vs 4.0 (range 0.27–13.3); p = 0.60) (Supplemental Figure S17) mRNA expression in the high dose BiAP group when compared to the remaining combined CPB/DHCA groups. Interestingly, a substantial difference was seen on immunohistochemistry staining for tissue NGAL. CPB/DHCA animals without high dose BiAP frequently demonstrated prominent NGAL staining that was restricted to the remaining healthy appearing tubules (Fig. 8A,B). Conversely kidneys from the high dose AP group and anesthesia controls demonstrated no significant NGAL staining (Fig. 8C,D).

## Discussion

### Key findings

We developed a novel large animal model of infant peripheral CPB/DHCA that produces clinically relevant AKI in the early post-CPB period. In this model, CPB/DHCA resulted in decreased serum and renal tissue AP activity. We found that these deficits in serum and tissue AP activity were corrected only in our highest dose BiAP infusion group (initial bolus of 75U/kg followed by an infusion of 25U/kg/hr). Preliminary efficacy data demonstrated a trend towards decreased proximal tubular injury in animals treated with high dose BiAP compared to untreated animals or animals receiving lower doses of BiAP. Kidney injury in the high dose BiAP group was comparable to anesthesia-only controls.

### Novel aspects of model development

To our knowledge, this study demonstrates the first use of a peripheral cannulation technique in an infant piglet model of CPB or DHCA-induced AKI. Multiple groups have published neonatal or infant swine models of CPB +/− DHCA utilizing central cannulation techniques^41–46^. Central cannulation has the advantage of larger venous cannula size, allowing faster and more complete venous drainage, as well as the ability to directly arrest the heart with the use of aortic cross-clamping and cardioplegia. The primary disadvantage of central cannulation is the high level of surgical expertise needed by the investigator team, potentially limiting feasibility of these large animal models as well as increasing cost in many cases.

Patel, *et al*. previously described the use of a minimally invasive cannulation strategy for CPB in adult pigs using the right internal carotid artery and right external jugular vein^47^. We found this approach to be straightforward in infant piglets and conducive to adequate venous drainage for cooling on CPB. In addition, we found that DHCA could consistently be achieved using this peripheral approach without the use of aortic cross-clamping and cardioplegia. Our animals were able to separate from bypass after rewarming with a level of heart failure/inotropic support similar to that of infants undergoing cardiac surgery for the repair or palliation of CHD. Overall, a peripheral cannulation strategy proved to be highly feasible in infant animals and we believe it represents an attractive alternative to central cannulation, particularly for the study of non-cardiac multi-organ injury post-CPB/DHCA.

### Importance of early proximal tubular injury

Our model produces histologic findings consistent with injury to the proximal tubules including epithelial thinning with bleb formation, tubular dilation, and brush border loss. These histologic findings of acute tubular injury are complemented by increased urinary NGAL/Cr ratio and NGAL mRNA expression in CPB/DHCA animals, but as expected no difference in serum creatinine. While serum creatinine and urine output represent the standards for clinical diagnosis of AKI given the inability to routinely measure injury directly through biopsy, the rise in serum creatinine is a late finding, generally occurring hours to days after the initial injury^11,12,48^. Additionally, interpretation of a rise in serum creatinine can be quite complicated in neonates and infants undergoing cardiac surgery due to fluid overload, differences in muscle mass, the presence of maternal creatinine, and physiologic changes in glomerular filtration rate^49^.

Recent research points towards the importance of early proximal tubular injury as a critical step prior to the subsequent development of renal dysfunction^11,12,14,20,21,50^. Biomarker evidence of proximal tubular injury appears in children as early as 2 hours after the initiation of cardiac surgery with CPB, even earlier than the time point used in our study^11,14,20^. In an elegant study based on recommendations of the Acute Dialysis Quality Initiative Consensus Conference, Basu *et al*. examined the  relative importance of early kidney dysfunction (measured by 2-hour plasma cystatin C) versus early tubular injury (measured by 2-hour urine NGAL) in the evolution to subsequent severe clinical AKI in pediatric cardiac surgery patients^15^. Patients were divided into 4 categories based on these early biomarker assessments: 1) no evidence of injury or dysfunction, 2) dysfunction without injury, 3) injury without dysfunction, or 4) combined dysfunction and injury. Early dysfunction alone (elevated cystatin C) demonstrated poor sensitivity and specificity for identifying patients who would ultimately develop AKI. This early dysfunction was hypothesized to represent a transient or physiologic change in glomerular filtration without damage to the tubules themselves. Presence of biomarker evidence of tubular injury (elevated NGAL), however, was highly specific (98%) for the subsequent development of severe AKI although with low sensitivity (potentially related to the finding in our model that the most severely injured tubules do not demonstrate significant NGAL staining on immunohistochemistry). The finding of combined early injury and dysfunction maintained high specificity with modestly increased sensitivity. Collectively these findings support the use of proximal tubular injury as an important early renal outcome measure following pediatric cardiac surgery.

### Animal models of CPB-induced AKI

Our findings of proximal tubular injury are consistent with previously published porcine models of CPB +/− DHCA induced AKI^13,42,44,47,51^. Wang, *et al*. found similar qualitative (heterogeneous proximal tubular dilation and epithelial flattening) and quantitative histologic changes (mean score 3.2 in CPB animals versus 0.33 in anesthesia controls) at 2 hours post-CPB in their neonatal piglet model of CPB without DHCA, utilizing the previously described low magnification scoring system^42^. Tirilomis *et al*. also described this pattern of proximal tubular injury in neonatal piglets 6 hours after CPB compared to minimal histologic changes in the kidneys of piglets undergoing sham sternotomy and laparotomy^44^. Previous adult porcine models using either central^13^ or peripheral^47,51^ cannulation techniques also demonstrated consistent but heterogeneous early proximal tubular injury following CPB. Taken together, our study and others demonstrate a highly reproducible pattern of early, heterogeneous renal cortical injury in swine of diverse ages undergoing variations of CPB with or without DHCA. Furthermore, we and others do not find evidence of excessive injury (complete cortical necrosis or severe early acute tubular necrosis) or substantial inflammatory cell infiltrate with these early injury models^13,47,51^. Although the paucity of human renal histology from the early post-CPB period makes direct comparison to porcine models challenging, a single study of ischemic injury in renal transplant grafts demonstrated similar tubular injury^52^ and we believe that the level of proximal tubular injury present in these models is consistent with the common finding of biomarker evidence of proximal tubular injury seen in adults and children following cardiac surgery^11,12,14,20,21,50^.

From a biomarker perspective our model demonstrated a mix of expected and unexpected findings. Our most expected finding was an early increase in serum and urine NGAL as well as tissue NGAL mRNA expression in animals exposed to CPB/DHCA. NGAL is a 25 kDa protein typically expressed at low levels in multiple tissues including kidney^20^. Early in the course of ischemia/reperfusion injury NGAL renal expression increases, particularly in the proximal tubule as demonstrated by a series of experiments in rodents^50^. This increased expression leads to elevated urine and serum levels within an hour after CPB, although interpretation of measurements is complicated by alternative sources of NGAL (notably neutrophil production) as well as filtration and reabsorption of soluble NGAL^53^. While data concerning NGAL and AKI in porcine models of kidney ischemia-reperfusion are relatively rare, Goebel, *et al*. have demonstrated increased serum NGAL in adult pigs as early as 2 hours after CPB. Similarly, Mei, *et al*. showed a rise in both serum and urine NGAL peaking at 6 hours after ischemia-reperfusion injury in their porcine model of ventricular fibrillation^54^. In contrast, human data on urine and serum NGAL levels after CPB are robust and demonstrate consistent increases in both serum and urine NGAL following CPB, especially in neonates and infants undergoing repair of CHD^12,20,55^.

Our model also demonstrated two unexpected, novel findings regarding biomarkers of AKI. First, we found that the majority of tissue NGAL staining occurred in otherwise healthy appearing proximal tubules, with progressive loss of staining in tubules with histologic evidence of injury. Similarly, there was a non-significant trend towards higher NGAL mRNA expression in mild to moderately injured kidneys compared to either healthy kidneys or severely injured kidneys. To our knowledge, this pattern of NGAL expression has not previously been described, although Pedersen, *et al*. did find decreased urinary NGAL concentrations with increased length of ischemic time in a porcine model of unilateral renal ischemia^53^. These findings support the idea that NGAL expression may occur primarily in stressed but not highly injured proximal tubules and could help explain some of the inconsistency in NGAL prediction of subsequent AKI seen in recent multicenter clinical studies^15,21^.

Our second unexpected finding was the consistent suppression of KIM-1 mRNA expression in injured kidneys early after CPB/DHCA. KIM-1 is a cell membrane glycoprotein that is highly expressed in the kidney 12–24 hours after ischemic renal injury in rodent models^56–58^ and humans^11,57^. While the exact function of KIM-1 is only partially understood, studies have demonstrated increased levels in both regenerating proximal tubular cells and cellular debris late after ischemic injury, suggesting a role in both cellular regeneration and potentially as a marker for phagocytosis of dead cells^56,57^. Most studies report minimal KIM-1 expression in healthy kidneys^11,56–58^ and little is known about tissue expression of KIM-1 early after ischemia-reperfusion injury. To our knowledge ours is the first report of depressed KIM-1 mRNA expression in injured kidneys in the early post-ischemic period; additional studies are warranted to understand the physiologic significance of this finding.

### Serum and renal AP activity post-CPB

Observational cohort studies in adults^35,36^ and children^7,33,37^ undergoing cardiac surgery have consistently demonstrated decreased serum AP activity in the immediate postoperative period. Previous studies in our center point towards a combination of early depletion secondary to low AP content in the blood prime of the CPB circuit (unpublished data) as well as ongoing loss of AP activity in the postoperative period^37^. The etiology of this ongoing loss in humans is not fully understood. It has been suggested that during the process of dephosphorylation, AP may form complexes with pathogen or damage associated molecular pattern molecules, which are subsequently cleared by endothelial cells and macrophages leading to decreased circulating AP^59^, however other studies including those from our group have shown dephosphorylation of key pathologic molecules (particularly adenine nucleotides) without complex formation^24,60,61^. Similar to findings in humans, our model produced an early decrease in serum AP activity after initiation of CPB due to depletion of AP with introduction of the low-AP activity blood prime, and persistently low activity through rewarming. Serum AP activity in our animals then gradually increased through 4 hours post-CPB/DHCA, a finding similar to that in adult patients^36^ but different from the more prolonged deficiency seen in our pediatric patients. As our study was not designed to explore the mechanism of ongoing AP depletion, further studies are required to understand if the prolonged deficiency in pediatric patients is secondary to ongoing consumption of AP in the face of persistent post-operative pathophysiology or decreased capacity to produce new AP compared to adult humans or our infant piglets.

To our knowledge the effects of CPB/DHCA on kidney tissue AP activity have not been previously described. We found decreased tissue AP activity after CPB/DHCA, especially in kidneys with histologic evidence of proximal tubular injury. Heinert, *et al*. demonstrated marked decrease in the renal tissue AP activity from biopsies of human kidneys with a variety of pathophysiology including chronic ischemia^62^. In the same study, the authors also demonstrated decreased renal tissue AP activity in rats with ischemia reperfusion injury^62^. Other groups have demonstrated similar findings in rodent models including a dose-response of lower renal tissue AP activity with longer periods of ischemia^63,64^. This decrease in AP activity is thought to be secondary to loss of brush border AP during proximal tubular injury. Decreased tissue AP activity has also been reported in an animal model of intestinal ischemia/reperfusion, again due to loss of brush border AP^65^, suggesting a common response of multiple epithelial-lined abdominal organs to ischemia-reperfusion injury. Interestingly, rodent models of renal endotoxin exposure result in increased renal tissue AP expression^66^, demonstrating that tissue AP may have different responses depending on the mechanism and severity of renal injury^67^.

### AP therapy: dosing and preliminary efficacy

As the final goal of our study, we sought to increase AP activity in both serum and renal tissue using escalating doses of BiAP infusion as well as gather preliminary efficacy data on the incidence and severity of post-CPB/DCHA AKI. We found that only our highest dose of BiAP (75U/kg bolus followed by and infusion of 25U/kg/hr) significantly increased serum AP activity by a factor of ~5 fold above baseline. This dose is similar to the regimen used by Su, *et al*. to reduce inflammation and improve pulmonary function (without apparent adverse effects) in a sheep model of sepsis^68^. The high dose BiAP infusion also was the only dose to result in increased renal tissue AP activity in our model. This finding is consistent with prior distribution studies performed using a human recombinant AP molecule, where injected AP was preferentially delivered to the blood and liver with lower doses achieved in the kidney and other abdominal organs, therefore requiring higher serum levels to result in increased renal tissue levels^69^.

From an efficacy standpoint, we found a trend towards decreased proximal tubular injury in animals treated with the highest dose BiAP infusion. To our knowledge this study is the first to evaluate AP therapy in an animal model of CPB with or without DHCA, although rodent models of isolated renal ischemia-reperfusion injury have suggested a decrease in tubular injury with AP therapy^70^. Since there are no published studies to provide an estimate for the effect size of AP for the prevention post-CPB/DHCA induced AKI, we did not expect this dose-finding study to be adequately powered to establish efficacy. Instead, these results will be used to power future studies aimed at establishing potential efficacy of BiAP for prevention of AKI in this piglet model of infant CPB/DHCA.

### Future directions: AP therapy for the prevention of AKI in humans

Three recent phase 2 studies have demonstrated promising findings regarding the use of either BiAP or recombinant human AP for the treatment of sepsis-induced AKI^30–32^. The first two studies utilized BiAP, with a similar bolus dose (67.5U/kg) but a lower infusion rate (5.5U/kg/hr) compared to our compared to our study^30,31^. This dosing resulted in an increase in creatinine clearance as well as a decrease in cytokines and urinary AKI biomarkers without any reported adverse effects^31^. Serum AP activity was not tracked in either study as a means to assess successful drug delivery or dose response. In the largest study to date, Pickkers, *et al*. demonstrated a non-significant increase in creatinine clearance in the first 7 days of treatment (primary outcome) and significant increases in day 21 and 28 creatinine clearance (secondary outcomes) in subjects treated with recombinant human AP compared to placebo^32^. AP-treated subjects also demonstrated lower mortality in this study (secondary outcome). While not definitive, these studies likely support further evaluation in the form of adequately powered phase 3 clinical trials for sepsis-induced AKI. To date, no study has utilized any form of AP to attempt to reduce AKI after cardiac surgery. The direct translation of any protective effects of AP infusion in sepsis-induced AKI to cardiac surgery-induced AKI is inappropriate given the significant differences in timing and physiologic mechanisms of injury. However, it is possible that CPB-induced AKI may be more amenable to treatment than sepsis-induced AKI, as the timing of injury is known and AP could be delivered as prophylaxis prior to initiation of injury rather than as treatment following development of AKI. With this goal in mind, future studies should focus on firmly establishing the efficacy of BiAP infusion to prevent AKI in large animal models of CPB with or without DHCA and, if warranted, phase 1 clinical trials in at-risk cardiac surgery populations.

### Limitations

The primary limitation of our study is the relatively small number of animals in each experimental group in the setting of heterogeneous kidney injury. While small animal models of ischemic AKI utilizing renal pedicle clamping produce more consistent levels of AKI, it has generally been difficult to translate these findings to humans^71,72^. Large animal models produce more heterogeneous injury^13^ that is similar to human disease^52^, but may require larger numbers of animals to demonstrate efficacy of novel therapeutic agents. In an attempt to adhere to guidelines advocating for the reduction the number of animals used to achieve a scientific goal, we chose as a first step to identify the optimal dose from a delivery standpoint to correct the underlying deficiency of AP after CPB/DHCA. We therefore designed our project as a dose finding study with the primary goals of identifying the level of injury in the model, the biochemical changes in the molecule of interest (AP), and the ability to correct this biochemical change with increasing doses of BiAP (the equivalent of a phase 1 study in humans). The study also generated preliminary efficacy data with which to appropriately power a subsequent larger study using the optimal dose identified from this current work.

Additional limitations include the use of a single early time point for the determination of tissue-level injury and biochemistry. Although AKI is thought to occur early after infant cardiac surgery, injury likely continues to evolve in the first 12–24 hours post-operatively. Future studies should incorporate longer post-CPB observation times with the use of serial noninvasive biomarkers to help track injury throughout the post-CPB period, ultimately to include survival models. Also, as we and others have identified, the natural history of renal biomarkers as well as their mechanistic link to tissue damage remain incompletely defined in both large animal models and humans. The field would greatly benefit from mechanistic natural history studies of AKI throughout the post-CPB period to further expand our understanding of noninvasive markers of AKI. In this study, we also focused on injury to a single (although important) organ. Post-CPB/DHCA injury is a multi-system problem and future research should continue to explore the integrated systems biology of this multi-organ injury as well as the systemic effects of BiAP therapy (efficacy and safety). The current study also was not designed to further define the mechanisms of action of AP in prevention of AKI beyond those already assessed in the scientific literature. Further studies are needed to evaluate the relative contributions of particular AP targets (most notably adenine nucleotides and endotoxin) to the kidney injury seen in our model. Finally, to minimize variability in this early pre-clinical study, we chose to focus on a single sex with a relatively narrow weight range. Additional studies are needed to establish the effects of sex, age, and weight on both the biology of post-CPB/DHCA injury as well as the pharmacology and efficacy of BiAP infusion for the prevention of AKI in this setting.

## Methods

### CPB/DHCA piglet model

The animal protocol used in our studies was approved by the Institutional Animal Care and Use Committee of the University of Colorado Anschutz Medical Campus in accordance with the Guide for the Care and Use of Laboratory Animals (National Institutes of Health) and the ARRIVE guidelines. Under isoflurane anesthesia, infant female pigs (5–10 kg) underwent peripheral CPB through cannulation of the internal carotid artery and external jugular vein using a 10fr DLP pediatric arterial cannula and 14fr Bio-Medicus one-piece venous cannula (Medtronic, Minneapolis, USA). Cannulation was performed after standard surgical dissection of the vessels via a right neck approach and ligation of the cranial end of each vessel. After gaining caudal control of each vessel, the vessel was incised and dilated, followed by cannula placement. The external jugular vein was selected for this model due to its large size in our animals (generally larger than the internal jugular vein) and distance from the internal carotid, which simplified subsequent placement of the arterial cannula.

The CPB circuit consisted of a D101 pediatric oxygenator (LivaNova PLC, London, UK), low volume tubing, and a standard roller pump. The animals were cooled using the CPB circuit to 22 °C rectal temperature to induce circulatory arrest without the need for aortic cross-clamp and cardioplegia. Once the target temperature was reached with accompanying asystole, CPB was stopped and the animals were left in DHCA for 75 min. Following the period of DHCA, CPB was restarted and the animals were rewarmed over ~30 min to 36 °C. Inotropic support was initiated at 34 °C using a standardized starting dose of epinephrine, dopamine, and milrinone at 0.05 µg/kg/min, 2 µg/kg/min, and 0.5 µg/kg/min respectively. The piglets were then separated from CPB and provided ICU care for 4hrs including full mechanical ventilation and inotropic/vasoactive support (titrated to a mean arterial pressure of 45–65 mmHg including addition of vasopressin infusion as needed). An additional group of animals were ventilated under anesthesia for 7hrs without undergoing CPB/DHCA to serve as anesthesia controls.

### BiAP infusion

Five animals underwent the CPB/DHCA protocol without BiAP infusion to serve as the baseline model. Medical grade BiAP (bRESCAP, Alloksys, The Netherlands) was then given to three groups of piglets (n = 5 per group) in escalating doses to assess the effect on serum AP activity and identify the best candidate dose for reduction of AKI in this model. BiAP was administered as a bolus prior to initiation of CPB along with simultaneous initiation of a continuous infusion via a femoral central venous catheter. The infusion was continued through euthanasia. Dosing was provided at the following levels: low (3U/kg bolus followed by 1U/kg/hr infusion); medium (15U/kg bolus followed by 5U/kg/hr infusion); high (75U/kg bolus followed by 25U/kg/hr infusion). Doses were chosen base on extrapolation from phase 2 human sepsis studies (15U/kg bolus followed by 5U/kg/hr infusion-moderate dose group) as well as 5-fold lower and higher doses^67^.

### Clinical monitoring and lactate measurements

All animals underwent continuous cardiovascular monitoring including femoral arterial blood pressure, central venous pressure, and electrocardiographic monitoring. Heart rate and blood pressure were recorded hourly along with doses of inotropic and inodilator medications and the combined VIS^39,40^. Fresh whole arterial blood was collected at cannulation, rewarming, and euthanasia to measure arterial lactate levels using iSTAT point of care testing (Abbott, Princeton, NJ).

### Serum and tissue AP activity measurements

Five ml of whole blood was collected from the femoral arterial line at induction, cannulation, rewarming and euthanasia and placed in a standard red top serum tube for processing. Once serum was separated it was frozen and stored at −70 °C for batch analysis. Serum AP activity was measured using a commercially available DRI-CHEM analyzer (HESKA Lab Systems, Loveland, CO). To determine kidney tissue AP activity, 50 mg of tissue was homogenized in 500 µl of AP assay buffer (Biovision, Milpitas, CA) then centrifuged at 11,000 × g for 20 min. Supernatant was diluted 1:20 and analyzed using DRI-CHEM analyzer using the same methods as performed for serum analysis.

### Histology

Kidney tissue was collected from each animal at euthanasia and fixed overnight in 10% formalin, paraffin-embedded, and sectioned at 4 µm. Sections were oriented to demonstrate a radial section of cortex and medulla. Serial sections were stained with hematoxylin-eosin (H&E) and evaluated for severity of kidney injury by a blinded board certified pediatric pathologist with a specialization in pediatric kidney disease (Dr. Treece) utilizing previously published methodology^13,42,47,52^. At low magnification (2X), sections were evaluated for tubular dilatation and epithelial flattening and scored 0–4 based on the percent of the parenchyma affected, as follows: 0 = none to less than 5%, 1 = 5–25%, 2 = 26–50%, 3 = 51–75%, 4 = greater than 75%.

Sections were also evaluated for NGAL by immunohistochemistry. After deparaffinization and rehydration, sections were blocked with 1.5% goat serum (Vector Laboratories, Burlingame, CA) in phosphate-buffered saline for 30 min, then incubated with rabbit polyclonal NGAL (1∶20; Cloud-Clone, Katy, TX) antibody overnight, washed with phosphate-buffered saline, and incubated with goat anti-rabbit biotinylated secondary antibody (1:200; Vector Laboratories) for 30 min. Vectastain Elite ABC reagent (Vector Laboratories) was then applied, followed by Nova red as substrate. Sections were counterstained with hematoxylin, dehydrated and cover-slipped.

### AKI serum and urine biomarkers

Serum and urine samples were taken immediately prior to euthanasia for measurement of serum NGAL and urine NGAL/creatinine ratios. Pig NGAL ELISA kit (Enzo Life Sciences, Farmingdale, NY) was used to analyze serum and urine samples by a Biotek plate reader (Winooski, VT). Creatinine assay kit (R&D Systems, Minneapolis, MN) was used to determine urine concentrations by Biotek plate reader (Winooski, VT). Serum creatinine was measured using a commercially available DRI-CHEM analyzer (HESKA Lab Systems, Loveland, CO).

### RNA preparation, RT, and real-time PCR

Total RNA was isolated from kidney tissue (snap frozen in liquid N_2_) using the RNeasy Mini Kit (Qiagen, Santa Clarita, CA) as described in the manufacturer’s protocol. RNA concentrations were quantified at 260 nm, and the purity and integrity were determined using a NanoDrop. RT and real-time PCR assays were performed to quantify steady-state mRNA levels of NGAL, kidney KIM-1, and IL-6. cDNA was synthesized from 0.5 µg of total RNA. Predeveloped TaqMan primers and probes (Applied Biosystems, Foster City, CA) were used for detection. Reporter dye emission was detected by an automated sequence detector combined with ABI Prism 7300 Real Time PCR System (Applied Biosystems, Foster City, CA). Real-time PCR quantification was performed with TaqMan b-actin controls and relative mRNA expression calculated using the 2^−ΔΔCT^ method.

### Statistics

Variables were expressed as means with standard deviations for normally distributed data and medians with ranges for skewed data. For multi-group comparisons, one-way ANOVA or Kruskal Wallis testing was initially performed (depending on the distribution) to assess for a difference among groups. If indicated, subsequent pairwise testing was performed using either Student’s t-test or Mann-Whitney U test, as appropriate for the data distribution, to determine specific differences between groups. P < 0.05 was considered statistically significant. GraphPad Prism 6 (La Jolla, CA) was used for data analyses and graphics production.

## Supplementary information

Supplementary Figures

## Data Availability

The full REDCap database with results for the current study is publically available at ResearchGate, https://www.researchgate.net/profile/Jesse_Davidson/research. Additional detailed datasets generated during and/or analyzed during the current study (including full ELISA and PCR outputs) are available from the corresponding author on reasonable request.
