# Supplementary material for: Alkaline Phosphatase Treatment of Acute Kidney Injury in an Infant Piglet Model of Cardiopulmonary Bypass with Deep Hypothermic Circulatory Arrest
Source: Sci Rep. 2019 Oct 2;9:14175. doi: 10.1038/s41598-019-50481-w (PMC6775126; doi:10.1038/s41598-019-50481-w)
Supplement: Supplementary file 1 — Supplementary Figures [file 41598_2019_50481_MOESM1_ESM.pdf]

Title: Alkaline Phosphatase Treatment of Acute Kidney Injury in an Infant Piglet Model of Cardiopulmonary Bypass with Deep Hypothermic Circulatory Arrest

Supplementary Figures

Authors: Jesse A. Davidson[1*], Ludmila Khailova[1], Amy Treece[2], Justin Robison[1], Danielle E. Soranno[1], James Jaggers[3], Richard J. Ing[4], Scott Lawson[5], Suzanne Osorio Lujan[1]

Institutions: [1]University of Colorado Denver, Department of Pediatrics; [2]University of Colorado Denver, Department of Pathology; [3]University of Colorado Denver, Department of Surgery; [4]University of Colorado Denver, Department of Anesthesiology; [5]Children's Hospital Colorado, Heart Institute

***Corresponding Author:**

Jesse A. Davidson, MD, MPH, MSc

Address: 13123 East 16th Ave, Box 100, Aurora, CO 80045

Phone: (303) 524-4709

Email: jesse.davidson@childrenscolorado.org

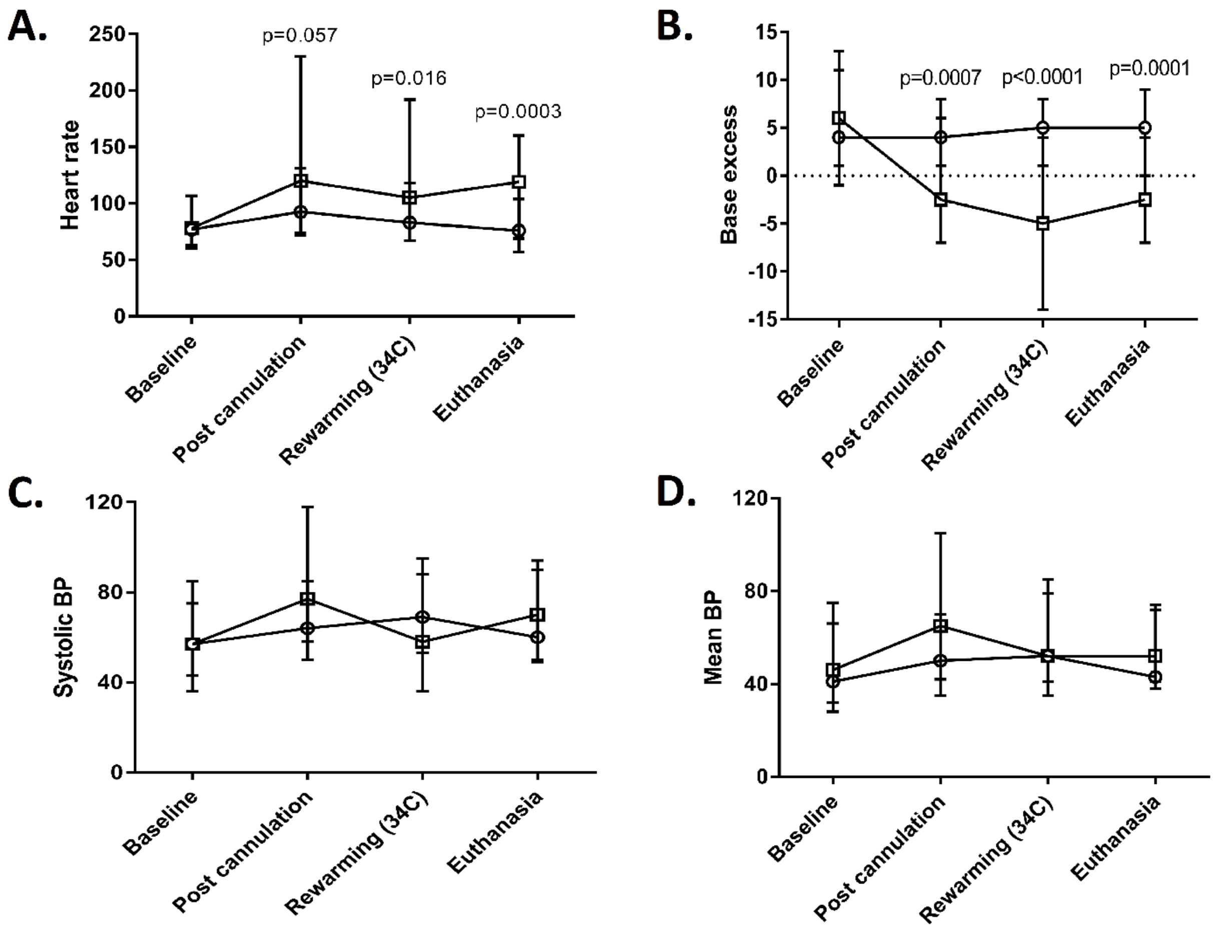

Supplemental Figure S1: Differences in physiologic parameters between animals undergoing CPB/DHCA compared to anesthesia controls. CPB/DHCA= cardiopulmonary bypass with deep hypothermic circulatory arrest

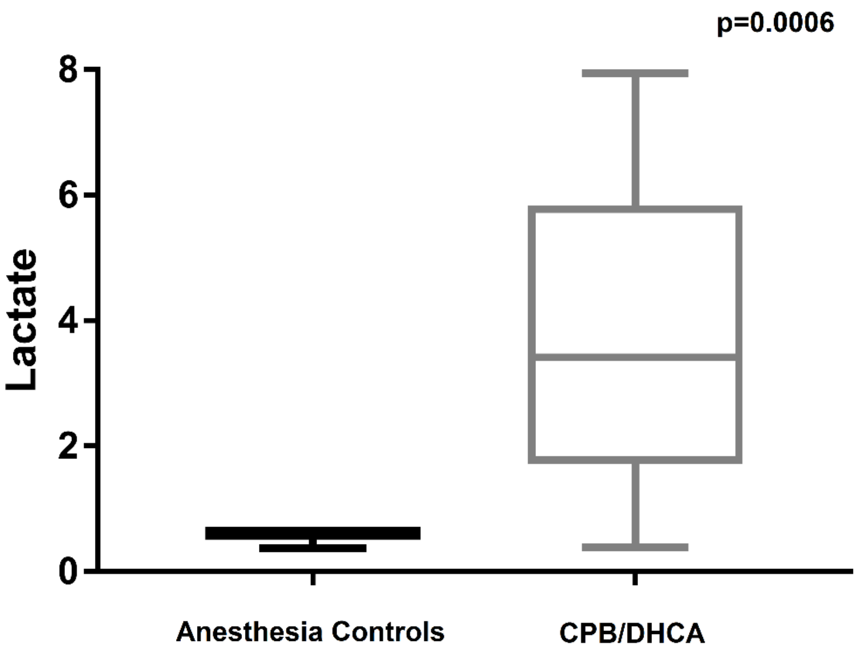

Supplemental Figure S2: Distribution of pre-euthanasia serum lactate levels in animals exposed to CPB/DHCA versus anesthesia-only controls. CPB/DHCA= cardiopulmonary bypass with deep hypothermic circulatory arrest

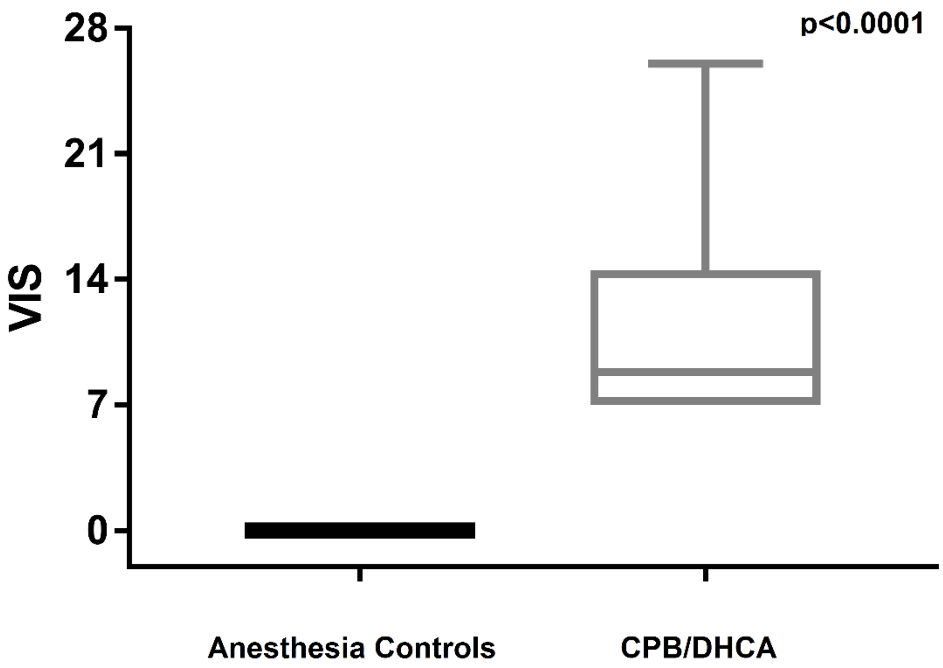

Supplemental Figure S3: Distribution of pre-euthanasia VIS in animals exposed to CPB/DHCA versus anesthesia-only controls. CPB/DHCA= cardiopulmonary bypass with deep hypothermic circulatory arrest; VIS=vasoactive inotropic score

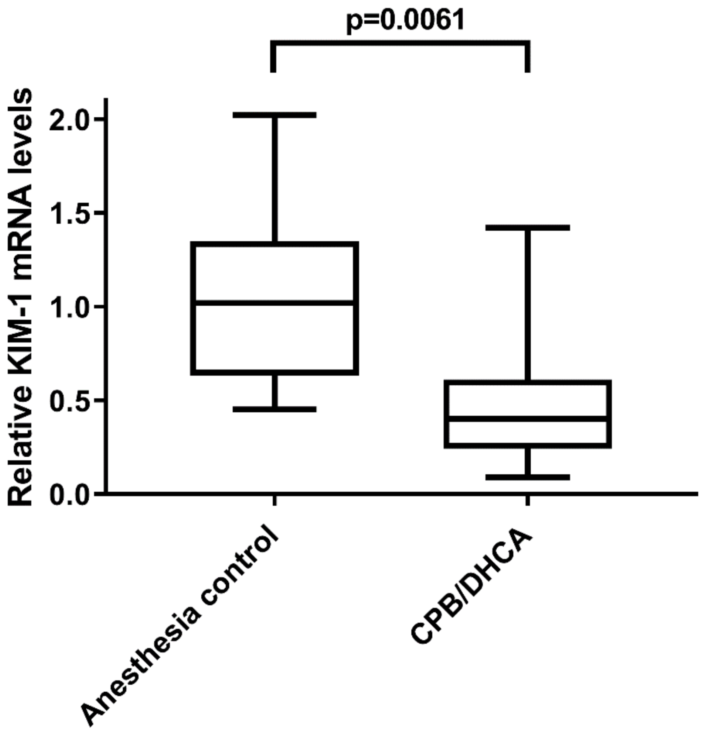

Supplemental Figure S4: Distribution of relative kidney tissue KIM-1 mRNA levels in animals exposed to CPB/DHCA versus anesthesia-only controls. CPB/DHCA= cardiopulmonary bypass with deep hypothermic circulatory arrest; KIM-1=kidney injury molecule-1

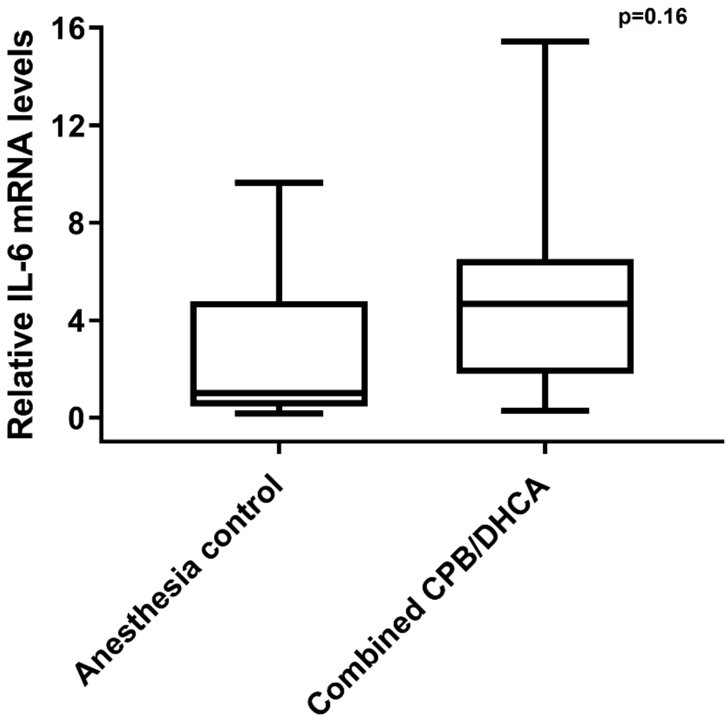

Supplemental Figure S5: Distribution of relative kidney tissue IL-6 mRNA levels in animals exposed to CPB/DHCA versus anesthesia-only controls. CPB/DHCA= cardiopulmonary bypass with deep hypothermic circulatory arrest; IL-6=interleukin-6

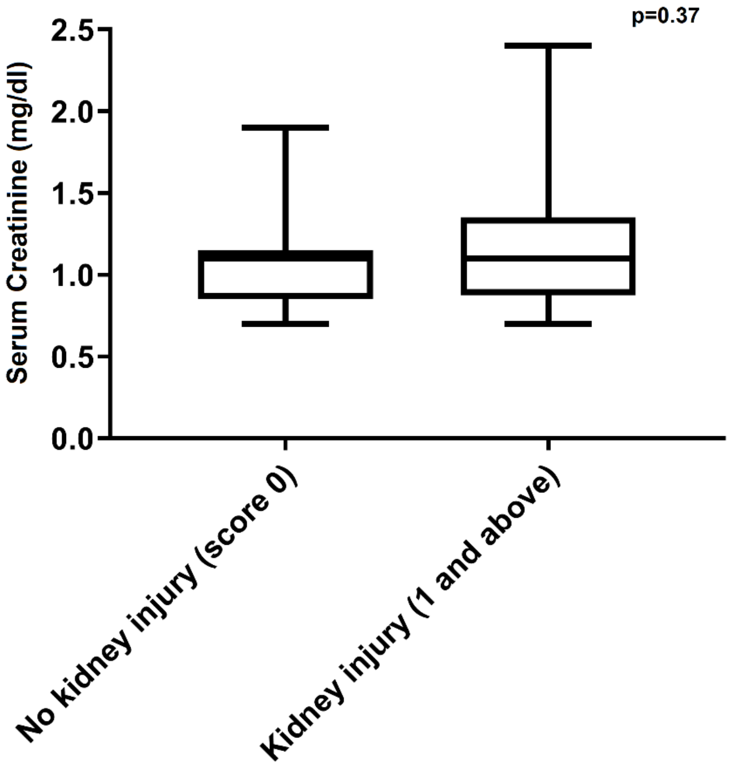

Supplemental Figure S6: Distribution of pre-euthanasia serum creatinine levels in animals with and without histologic evidence of acute kidney injury (kidney injury score 1-4 vs 0).

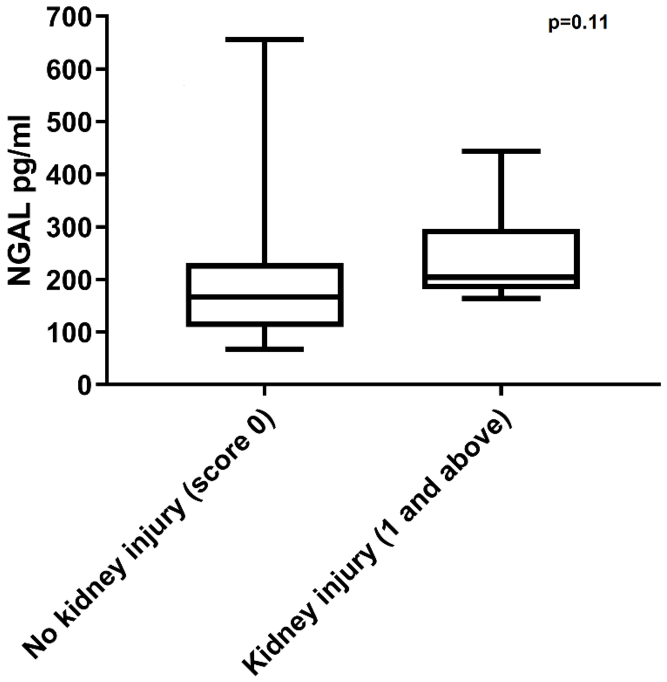

Supplemental Figure S7: Distribution of pre-euthanasia serum NGAL levels in animals with and without histologic evidence of acute kidney injury (kidney injury score 1-4 vs 0).

NGAL=neutrophil gelatinase-associated lipocalin

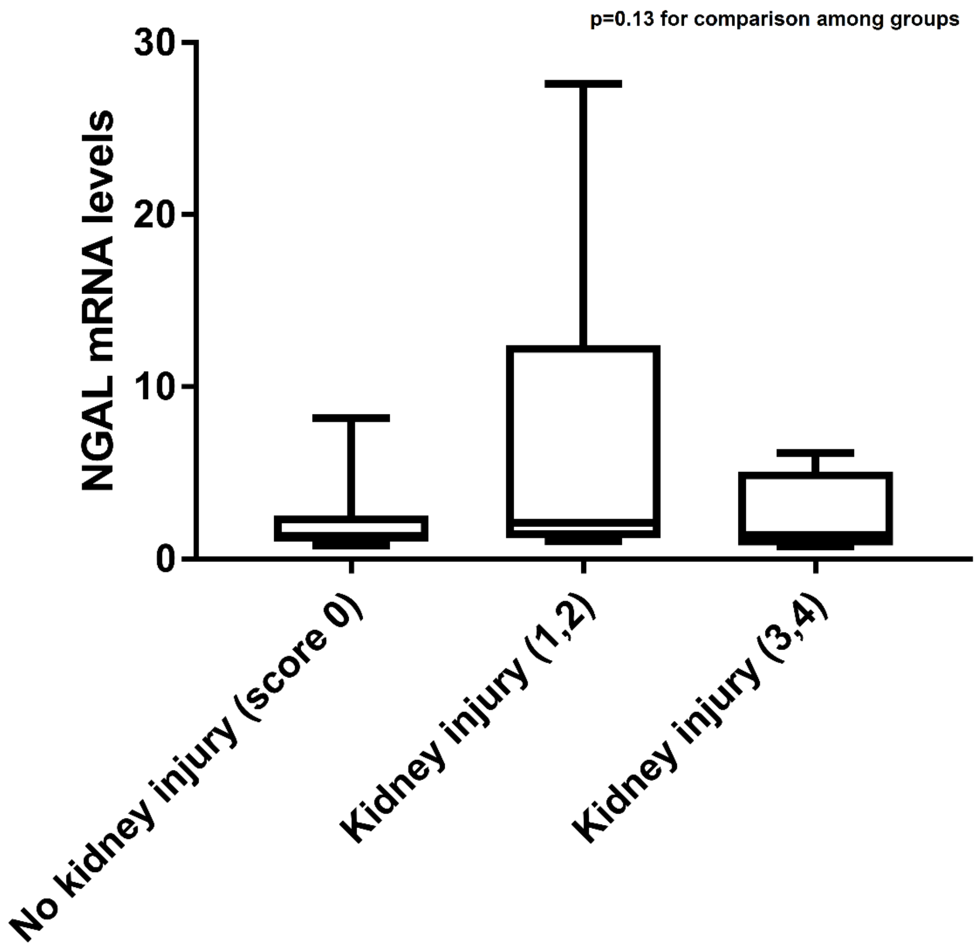

Supplemental Figure S8: Distribution of relative kidney tissue NGAL mRNA grouped by histologic kidney injury score (kidney injury score 0 vs 1-2 vs 3-4). P-value for Kruskal Wallis testing among multiple groups NGAL=neutrophil gelatinase-associated lipocalin

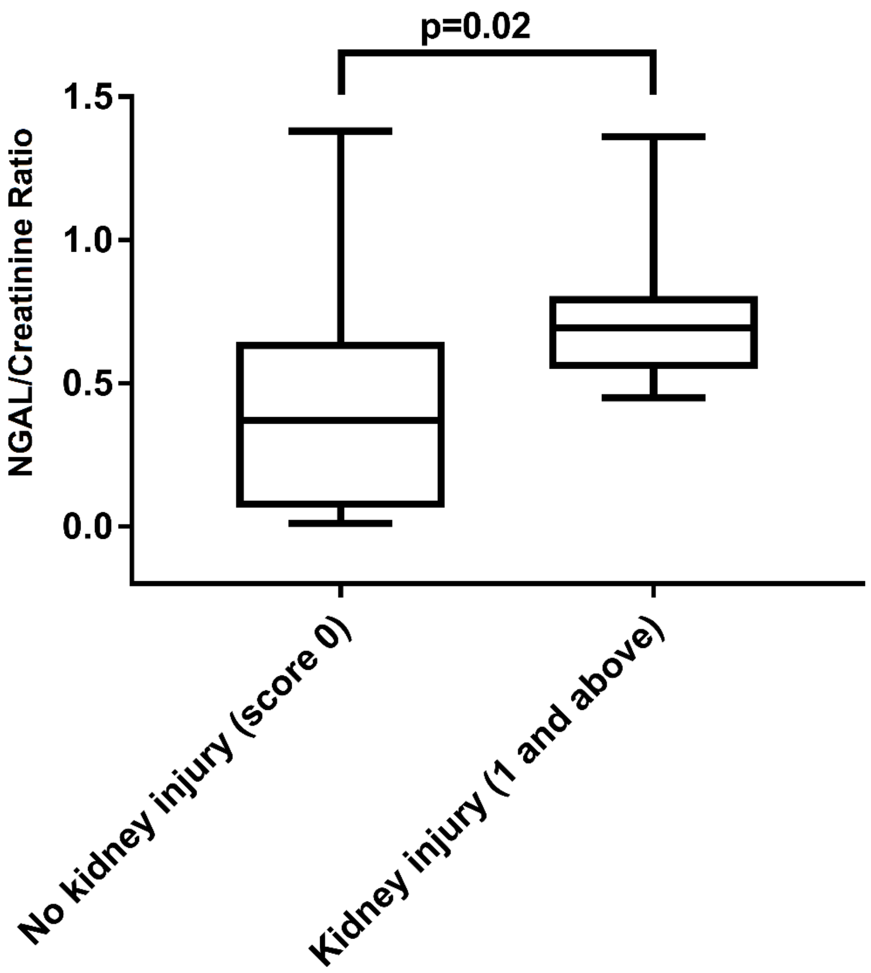

Supplemental Figure S9: Distribution of pre-euthanasia urine NGAL/creatinine ratios in animals with and without histologic evidence of acute kidney injury (kidney injury score 1-4 vs 0). NGAL=neutrophil gelatinase-associated lipocalin

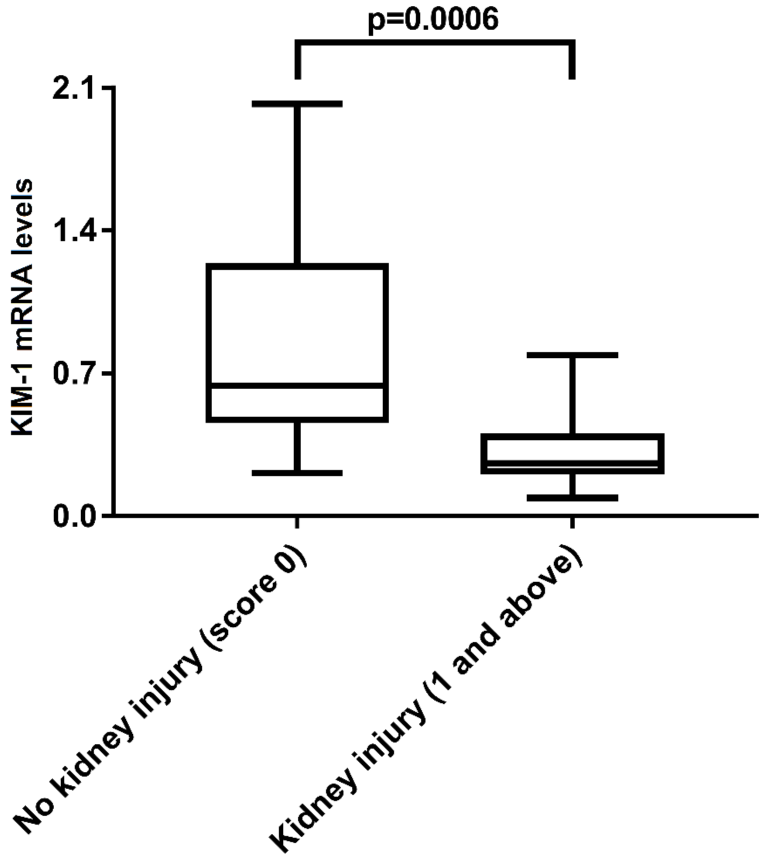

Supplemental Figure S10: Distribution of relative kidney tissue KIM-1 mRNA levels in animals with and without histologic evidence of acute kidney injury (kidney injury score 1-4 vs 0). KIM-1=kidney injury molecule-1

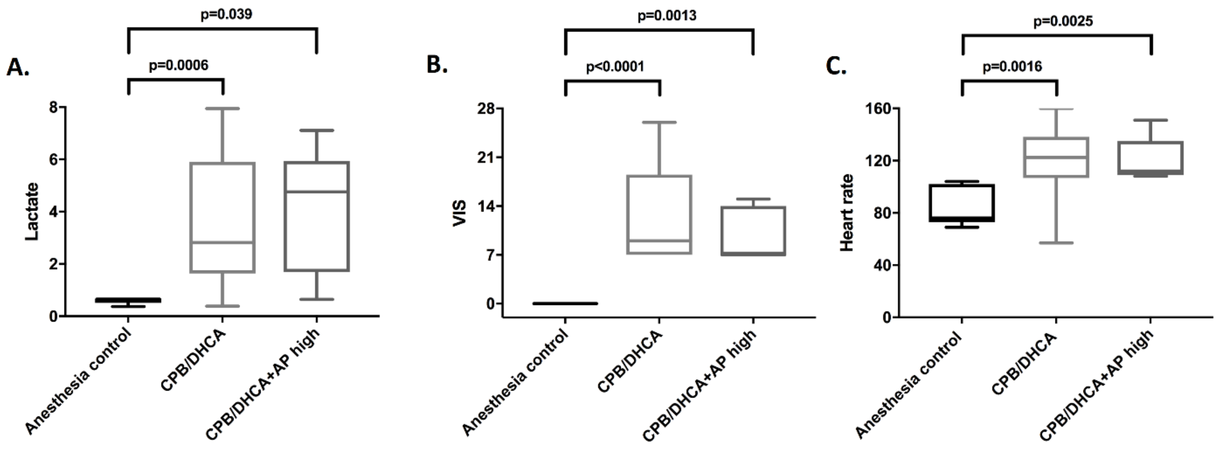

Supplemental Figure S11: Distributions of pre-euthanasia lactate, VIS, and heart rate in animals undergoing CPB/DHCA with high dose AP compared to the remaining CPB/DHCA animals or anesthesia controls. VIS=vasoactive inotropic score; CPB/DHCA= cardiopulmonary bypass with deep hypothermic circulatory arrest; AP=alkaline phosphatase

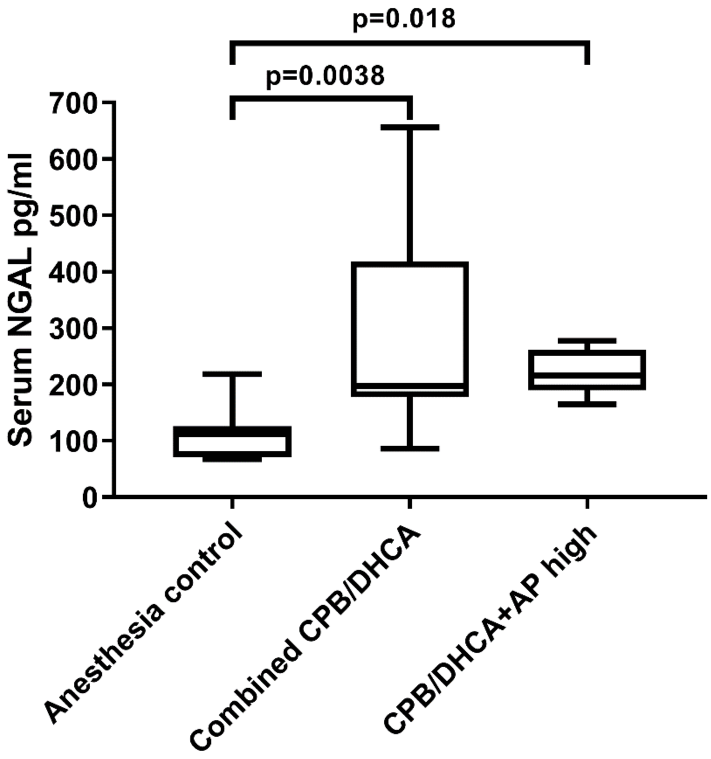

Supplemental Figure S12: Distribution of pre-euthanasia serum NGAL in animals undergoing CPB/DHCA with high dose AP compared to the remaining CPB/DHCA animals or anesthesia controls. NGAL= neutrophil gelatinase-associated lipocalin; CPB/DHCA= cardiopulmonary bypass with deep hypothermic circulatory arrest; AP=alkaline phosphatase

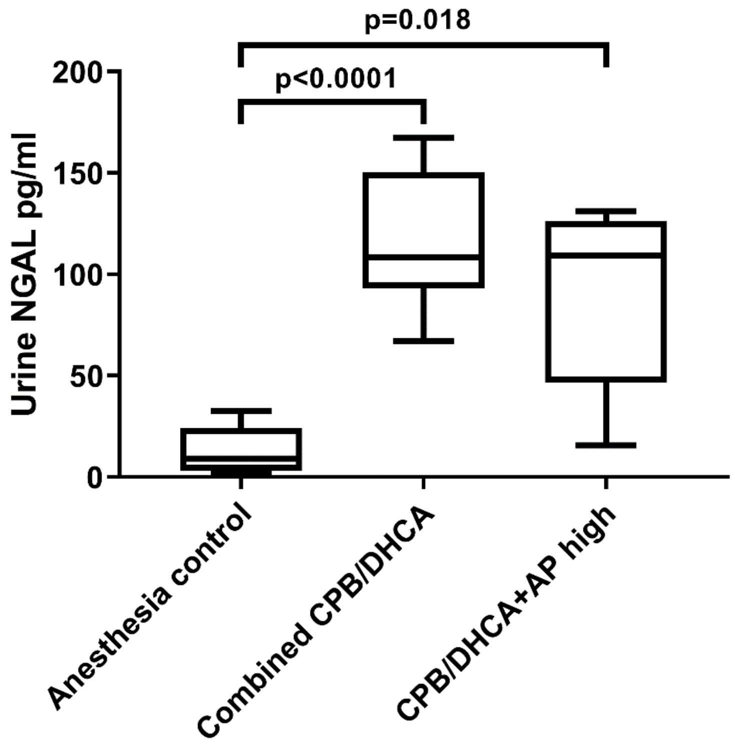

Supplemental Figure S13: Distribution of pre-euthanasia urine NGAL in animals undergoing CPB/DHCA with high dose AP compared to the remaining CPB/DHCA animals or anesthesia controls. NGAL= neutrophil gelatinase-associated lipocalin; CPB/DHCA= cardiopulmonary bypass with deep hypothermic circulatory arrest; AP=alkaline phosphatase

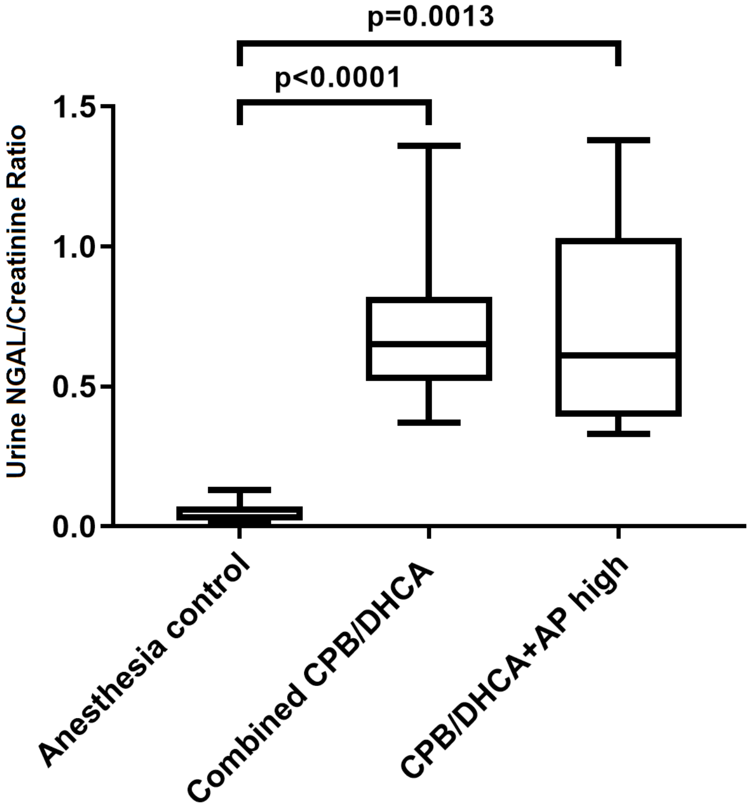

Supplemental Figure S14: Distribution of pre-euthanasia urine NGAL/creatinine ratios in animals undergoing CPB/DHCA with high dose AP compared to the remaining CPB/DHCA animals or anesthesia controls. NGAL= neutrophil gelatinase-associated lipocalin; CPB/DHCA= cardiopulmonary bypass with deep hypothermic circulatory arrest; AP=alkaline phosphatase

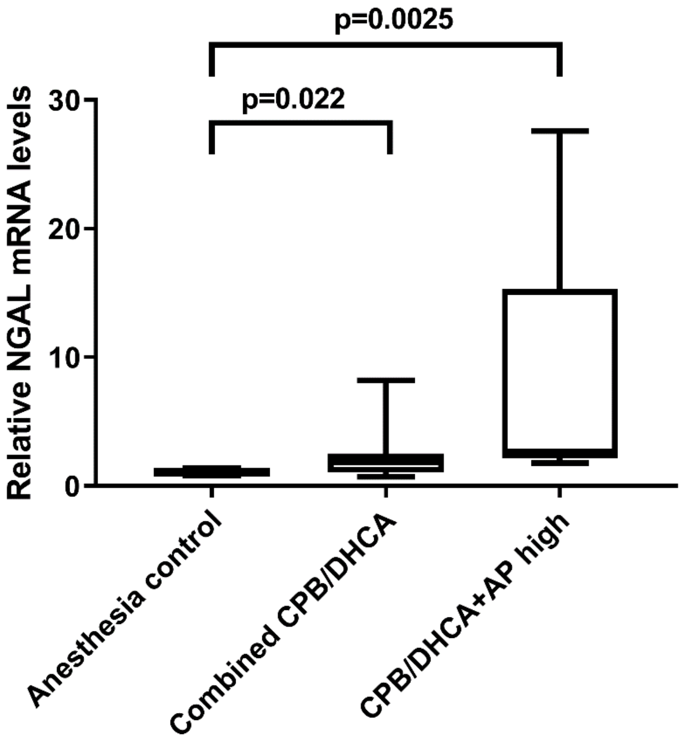

Supplemental Figure S15: Distribution of relative kidney tissue NGAL mRNA levels in animals undergoing CPB/DHCA with high dose AP compared to the remaining CPB/DHCA animals or anesthesia controls. NGAL= neutrophil gelatinase-associated lipocalin; CPB/DHCA= cardiopulmonary bypass with deep hypothermic circulatory arrest; AP=alkaline phosphatase

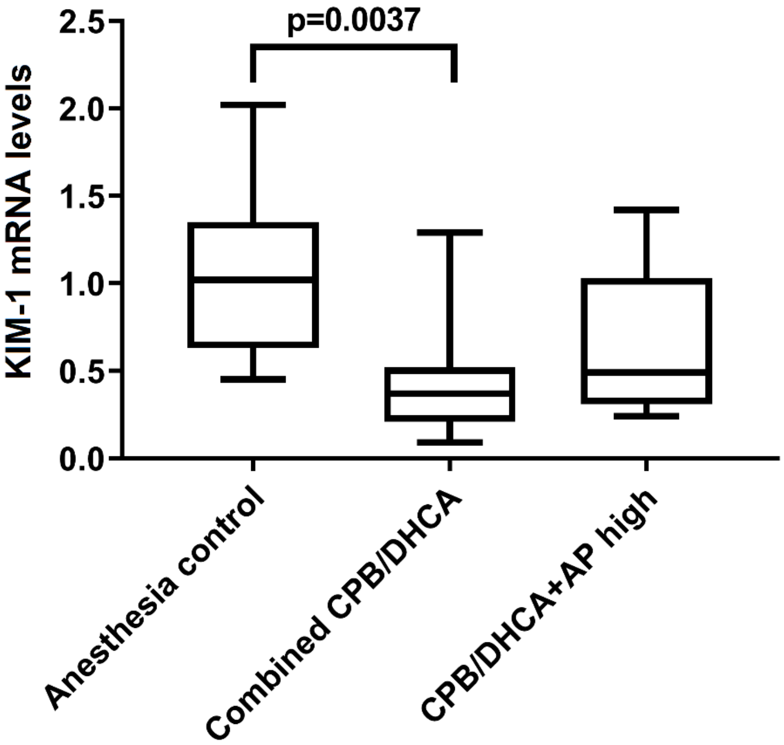

Supplemental Figure S16: Distribution of relative kidney tissue KIM-1 mRNA levels in animals undergoing CPB/DHCA with high dose AP compared to the remaining CPB/DHCA animals or anesthesia controls. KIM-1=kidney injury molecule-1; CPB/DHCA= cardiopulmonary bypass with deep hypothermic circulatory arrest; AP=alkaline phosphatase

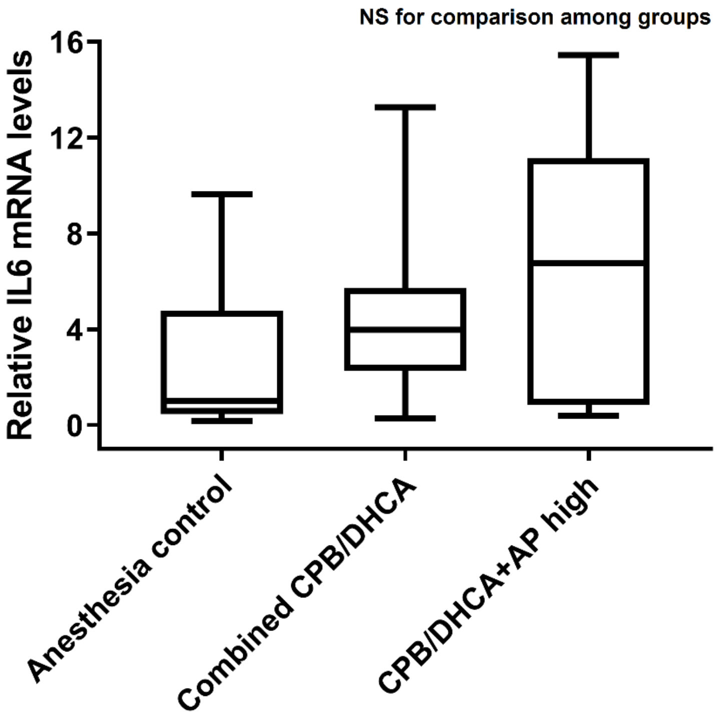

Supplemental Figure S17: Distribution of relative kidney tissue IL-6 mRNA levels in animals undergoing CPB/DHCA with high dose AP compared to the remaining CPB/DHCA animals or anesthesia controls. IL-6=interleukin-6; CPB/DHCA= cardiopulmonary bypass with deep hypothermic circulatory arrest; AP=alkaline phosphatase; NS=not significant on Kruskal Wallis testing for differences among groups
